# Evaluation of Knowledge and Attitude of Dental Hygienists and Dental Assistants Regarding People Living with HIV/AIDS and HIV-Associated Oral and Periodontal Lesions in Jeddah, Saudi Arabia

**DOI:** 10.7759/cureus.53719

**Published:** 2024-02-06

**Authors:** Shatha Bamashmous, Fatimah Almalki, Wehad Alrefaei, Eatizaz Alsamadani, Mohamed Fattouh, Laila M Kenawi, Eman Elfirt

**Affiliations:** 1 Department of Periodontology, Faculty of Dentistry, King Abdulaziz University, Jeddah, SAU; 2 Department of Dental Hygiene and Nursing, King Abdulaziz University Dental Hospital, Jeddah, SAU; 3 Department of Fixed Prosthodontics, Faculty of Dentistry, Cairo University, Cairo, EGY; 4 Department of Oral and Maxillofacial Surgery, Faculty of Dental Medicine, Umm Al-Qura University, Makkah, SAU; 5 Department of Endodontics, Faculty of Dentistry, Cairo University, Cairo, EGY; 6 Department of Restorative Dentistry, Faculty of Dental Medicine, Umm Al-Qura University, Makkah, SAU; 7 Department of Oral Medicine and Periodontology, Faculty of Dentistry, Cairo University, Cairo, EGY

**Keywords:** dental assistants, dental hygienists, aids, hiv, attitude, knowledge

## Abstract

Background: Oral manifestations serve as important indicators of human immunodeficiency virus (HIV) infection, and dental healthcare practitioners play a pivotal role in preventing and managing HIV. This study aims to assess and compare the knowledge and attitude of dental assistants and dental hygienists concerning people with HIV/acquired Immunodeficiency syndrome (AIDS) patients in Jeddah province of Saudi Arabia.

Materials and methods: This cross-sectional survey enrolled 160 dental hygienists and assistants practicing in Jeddah. Data was collected using a pretested, self-administered questionnaire comprising 50 questions that investigated knowledge about HIV/AIDS, awareness of HIV-associated oral and periodontal lesions, understanding of HIV transmission routes, and attitudes toward people with AIDS.

Results: In the study, 120 (75.0%) participants correctly recognized that individuals with HIV may appear healthy, while 123 (76.9%) participants admitted that HIV carriers have lower resistance to other diseases. Additionally, 126 (78.8%) participants confirmed an association between the virus and oral lesions, and 137 (85.6%) participants were aware of HIV transmission through blood. Moreover, 150 (93.7%) participants expressed their willingness to support, treat, and assist patients with AIDS, while only 10 (6.3%) participants expressed unwillingness. Notably, no statistically significant differences were found between dental assistants and hygienists in their knowledge and attitudes toward HIV/AIDS and people with AIDS.

Conclusion: This study demonstrated that dental hygienists and assistants possess good general knowledge regarding HIV/AIDS and are well-informed about the disease's transmission routes. Moreover, a significant majority endorses the importance of treating and supporting AIDS patients.

## Introduction

In the last decades, acquired immunodeficiency syndrome (AIDS), resulting from the human immunodeficiency virus (HIV), emerged as a significant global public health concern [1]. The rapid spread of this epidemic is due to improved life expectancy and a lack of knowledge regarding the mode of disease transmission [2]. World Health Organization (WHO) has reported that 40.4 million individuals have died of HIV, with approximately 39 million individuals living with HIV at the end of 2022 [3]. With ongoing disease transmission, about 1.3 million people acquired HIV globally in 2022 [3]. The prevalence of HIV in the Middle East and the Kingdom of Saudi is recognized as one of the lowest globally at 0.02% [4]. The first instance of HIV infection in Saudi Arabia was detected in 1984 [5], and the number of infections has been increasing gradually [6]. As of 2020, there were 12,000 adults and children living with HIV in Saudi Arabia [7]. In developed countries, disease transmission is often associated with drug abuse and homosexuality, while heterosexuality plays a crucial role in developing countries [8]. Discrimination and stigmatization in developing societies may be the main factors contributing to underreporting of cases [9].

The risk of infection by blood-borne pathogens such as HIV, hepatitis C (HCV), and hepatitis B (HBV) represents a significant threat to healthcare providers [10,11]. The likelihood of the virus transmission from a patient to a healthcare provider has been estimated to be 0.3% following a single percutaneous exposure to HIV-infected blood [12]. Since the implementation of highly active antiretroviral therapy (HAART), there has been a significant improvement in the life expectancy of individuals living with HIV [13]. Although the available medications are still not capable of eliminating the disease, they effectively halt its progression. High medication adherence significantly reduces the infectious potential of HIV-positive individuals to an extremely low level [14].

Oral lesions are observed in 30-80% of AIDS patients, and serve as a potential sign of deterioration in patients' general health, thereby presenting a significant global health concern [15]. With the rising numbers of patients with AIDS seeking dental care, dentists, dental hygienists, dental assistants, and dental students need to have a comprehensive understanding of the disease. Additionally, their attitude and behavior should align with the professional expectations [16].

Treatment and management of AIDS patients necessitate a cautious approach to protect dental professionals while preventing disease transmission [17,18]. Healthcare providers often face a high risk of occupational exposure to HIV infections in developing countries [19-21]. Dental healthcare personnel need to recognize that dealing with AIDS patients can be challenging, and the attitudes of dentists, dental hygienists, and assistants can significantly affect the process. Moreover, the dental staff’s fear of handling AIDS patients, and deficient knowledge in treating such patients may impact the outcomes of dental care [22].

The management of AIDS patients in dental clinics is a controversial subject and attitudes toward dental treatment vary across different countries, indicating the complexity of providing dental care for AIDS patients globally [23-26]. Moreover, several studies have assessed the level of knowledge among dental students regarding the treatment of AIDS patients [27-30]. While dental hygienists and assistants play a significant role in the dental treatment of HIV patients, only a few older studies are available that examine their knowledge and attitudes concerning HIV/AIDS. Almuzaini et al. compared the knowledge of dental assistants working at the university and within the Ministry of Health in Kuwait, finding that university dental assistants demonstrated superior knowledge [31]. However, the study identified several misconceptions that need to be addressed, along with noting negative behavior exhibited by some of the respondents. A study in Hungary revealed that only 5-10% of dental nurses had incorrect knowledge regarding the mode of transmission of HIV [32]. Another study carried out in Minnesota, United States, revealed that 76% of dental staff were uncomfortable managing and treating HIV patients in addition to 14% who had declined to treat them [33]. A survey involving dentists in California, United States, indicated that dental staff need greater knowledge and awareness regarding HIV and infection control measures [34]. Kitaura et al. conducted a study in Japan that showed that more than 90% of dental healthcare workers expressed a need for additional education on HIV, particularly on the mode of transmission and prevention of the disease [23]. In Tanzania, a study was carried out to evaluate the operating dental staff’s knowledge of HIV infection, with the results showing that 23% in 1988 and 26% in 1989 provided incorrect answers in response to the questionnaire [35].

Therefore, it is crucial to evaluate the knowledge of dental hygienists and assistants concerning HIV transmission to determine its adequacy and whether any interventions are necessary. Accordingly, the aim of the present study was to assess and compare HIV/AIDS-related knowledge, attitudes, and behavior among dental hygienists and dental assistants in Jeddah, Kingdom of Saudi Arabia. Additionally, the study aimed to identify potential areas for targeted education and training to improve the overall readiness and effectiveness of oral healthcare professionals in managing HIV/AIDS-related challenges.

## Materials and methods

Study design and participants

A cross-sectional questionnaire survey-based study was conducted among dental hygienists and assistants in Jeddah, Saudi Arabia. The study received ethical approval from the Ethics Committee at King Abdulaziz University Faculty of Dentistry, Jeddah, Saudi Arabia (approval number: 050-16). In the study, subjects were recruited using a convenience sampling technique, ensuring voluntary and anonymous participation. Inclusion criteria were dental hygienists and assistants practicing in private and public dental clinics, who had a license from the Saudi Commission for Health Specialty. They were invited to participate and signed a consent form indicating their agreement. Collected data were handled with strict confidentiality and utilized only for research purposes. Power calculations were conducted using the G*Power statistical power analysis program 3.1.9.4 (Franz Faul, University of Kiel, Kiel, Germany). To explore the correlation between dental hygienists’ and assistants’ knowledge of and attitude toward people living with HIV/AIDS, a sample size of 160 participants was determined by considering an alpha level of 0.05, a desired power of 0.9, and an effect size set at 0.25.

Study instrument

A self-administered questionnaire was used to collect data. The questionnaire was divided into five categories. The first section included demographic variables including age, gender, job role (hygienist or assistant), and years of experience. The second section focused on evaluating participants' knowledge concerning dental treatment for HIV/AIDS patients, comprising 10 questions with response options of "Yes", "No", and "I don't know". The maximum score achievable in this section was 30 points, with interpretations as follows: 0-10 points indicating a low level of knowledge, 11-20 points indicating a moderate level of knowledge, and 21-30 points indicating a high level of knowledge. The third section explored participants' understanding of HIV-associated oral and periodontal lesions, with 11 questions and response options of “Associated”, “Not associated”, and “I don’t know”. The maximum score attainable in this section was 33 points, with interpretations as follows: 0-11 points signifying a low level of knowledge, 12-23 points signifying a moderate level of knowledge, and 24-33 points signifying a high level of knowledge. The fourth section assessed knowledge of HIV/AIDS transmission routes through 17 questions with response options of "Yes", "No", and "I don't know." Scores in this section ranged from 0 to 51, again with interpretations as follows: 0-17 points indicating low-level knowledge, 18-34 points indicating moderate-level knowledge, and 35-51 points indicating high-level knowledge. Lastly, the fifth section assessed attitudes toward treating HIV-positive patients, featuring 12 questions categorized as "Agree" or "Disagree", with scores ranging from 0 to 24, with interpretations as follows: 0-12 points representing a negative attitude and 13-24 points indicating a positive attitude.

The survey questionnaire's validity and reliability were evaluated to ensure its suitability for collecting data. A pilot sample of 22 individuals from the study population was used for this purpose. To assess validity, Pearson's correlation coefficient was employed to measure the relationship between each questionnaire item and its respective dimension. Cronbach's alpha coefficient and the split-half technique were used to assess reliability. The results of the validity testing indicated that most of the questionnaire items exhibited statistically significant correlations with their respective dimensions suggesting that the survey questionnaire effectively collected the necessary data to achieve the research objectives. The reliability assessment showed a very high overall reliability with a Cronbach's alpha coefficient of 0.922, indicating a robust survey method suitable for data collection. Additionally, all dimensions showed high reliability exceeding the 0.70 threshold, suggesting the method's efficiency in data collection.

Statistical analysis

Statistical Analysis was performed using IBM SPSS Statistics for Windows, Version 21.0 (Released 2012; IBM Corp., Armonk, New York, United States). For nominal and categorical variables, simple descriptive statistics in the form of counts and percentages were employed to characterize the study variables. Conversely, continuous variables were represented by mean and standard deviation (SD). Mean scores and SD were calculated for each item within the knowledge and attitude domains. Pearson's correlation analysis was performed to assess the association between the overall scores of knowledge and attitude. An independent t-test was utilized to assess and compare the knowledge and attitudes of dental assistants and dental hygienists toward HIV/AIDS. Statistical significance was established when the calculated p-values from the analysis were less than 0.05.

## Results

Demographic data

A self-administered questionnaire was distributed to a random sample of 200 participants from the study population, yielding a response rate of 80% (n=160). A total of 160 eligible participants completed the questionnaire, ranging in age from 21 to 58 years, with an average age of approximately 31.34 ± 8.1 years. A significant majority, approximately 75.45%, were between 21 and 35 years of age. In terms of gender distribution, females constituted a substantial majority at 73.1% (117 individuals), with males representing approximately 26.9% (43 individuals) of the participants. The participants demonstrated a broad range of professional experience, extending from one to 24 years, with an average experience level of approximately 8.31 ± 6.13 years. Notably, most participants (approximately 50%) possessed a moderate level of professional experience, falling within the 3-12-year experience range. Regarding job roles, it was observed that hygienists constituted 31.3% of the participants (50 individuals), while the assistants comprised 68.7% of the participants (110 individuals).

Analysis of knowledge of and attitude toward HIV/AIDS

The results regarding general knowledge about HIV/AIDS, knowledge of HIV-associated oral and periodontal lesions, knowledge about routes of transmission of HIV, and attitudes toward patients with AIDS are presented in Tables 1-4.

**Table 1:** Participants' general knowledge about HIV/AIDS Data are presented as mean ± SD and n (%)

Item No.	Questions		Yes	No	I don’t know	Mean	SD
1	AIDS is caused by a virus	n	153	5	2	2.94	0.28
		%	95.6	3.1	1.3		
2	AIDS is contagious	n	124	17	19	2.66	0.68
		%	77.5	10.6	11.9		
3	AIDS is hereditary	n	34	112	14	2.13	0.53
		%	21.3	70.0	8.8		
4	There is a curative treatment for AIDS	n	41	105	14	2.17	0.56
		%	25.6	65.6	8.8		
5	An HIV-positive individual may look healthy	n	120	30	10	2.69	0.58
		%	75.0	18.8	6.2		
6	Resistance to other diseases is low	n	123	20	17	2.66	0.66
		%	76.9	12.5	10.6		
7	There is a vaccine for AIDS	n	18	118	24	1.96	0.51
		%	11.2	73.8	15.0		
8	Lymphocyte host defense cells are primarily affected by AIDS	n	95	10	55	2.25	0.94
		%	59.4	6.2	34.4		
9	Individuals with anti-HIV antibodies are HIV carriers	n	47	58	55	1.95	0.80
		%	29.4	36.3	34.4		
10	The average time interval between contracting HIV and production of antibodies is 6-12 weeks	n	51	18	91	1.75	0.91
		%	31.9	11.2	56.9		
Total score of knowledge about HIV/AIDS (mean ± SD)						23.16	2.79

**Table 2:** Participants' knowledge of HIV-associated oral and periodontal lesions Data are presented as mean ± SD and n (%)

Item No.	Questions		Associated	Not associated	I don’t know	Mean	SD
1	Oral Kaposi’s sarcoma	n	87	16	57	2.19	0.93
		%	54.4	10.0	35.6		
2	Oral candidiasis	n	126	16	18	2.68	0.67
		%	78.8	10.0	11.2		
3	Salivary gland enlargement	n	84	39	37	2.29	0.82
		%	52.5	24.4	23.1		
4	Xerostomia	n	90	33	37	2.33	0.83
		%	56.3	20.6	23.1		
5	Idiopathic thrombocytopenic purpura	n	42	25	93	1.68	0.86
		%	26.3	15.6	58.1		
6	Crohn’s disease	n	34	48	78	1.73	0.79
		%	21.2	30.0	48.8		
7	Herpes simplex virus	n	113	19	28	2.53	0.78
		%	70.6	11.9	17.5		
8	Necrotizing gingivitis	n	101	19	40	2.38	0.86
		%	63.1	11.9	25.0		
9	Non-Hodgkin’s lymphoma	n	46	37	77	1.81	0.86
		%	28.8	23.1	48.1		
10	Aphthous stomatitis	n	90	27	43	2.29	0.87
		%	56.2	16.9	26.9		
11	Aggressive periodontitis	n	92	30	38	2.34	0.84
		%	57.5	18.7	23.8		
Total score of knowledge of HIV-associated oral and periodontal lesions (mean ± SD)						24.25	5.98

**Table 3:** Participants’ knowledge about the routes of transmission of HIV/AIDS Data are presented as mean ± SD and n (%).

Item No.	Questions		Yes	No	I don’t know	Mean	SD
1	Sharing public toilets and swimming pools with a person infected with HIV/AIDS	n	50	104	6	2.28	0.53
		%	31.2	65.0	3.8		
2	Using an infected person’s items like comb, underwear, and towels	n	56	93	11	2.28	0.58
		%	35.0	58.1	6.9		
3	Sharing a razor blade with a person infected with HIV/AIDS	n	120	30	10	2.69	0.58
		%	75.0	18.8	6.2		
4	Contacting a person infected with HIV/AIDS (shaking hands)	n	27	123	10	2.11	0.47
		%	16.9	76.9	6.2		
5	Using food tools (spoon, fork, etc.) of a person infected with HIV/AIDS	n	64	82	14	2.31	0.63
		%	40.0	51.2	8.8		
6	Exposure to an infected person who coughs or spits	n	60	87	13	2.29	0.61
		%	37.5	54.4	8.1		
7	Mosquito bite	n	63	81	16	2.29	0.64
		%	39.4	50.6	10.0		
8	Sharing injection needles or surgical devices of a person infected with HIV/AIDS	n	137	18	5	2.83	0.46
		%	85.6	11.3	3.1		
9	An infected pregnant woman infecting her unborn baby	n	115	28	17	2.61	0.67
		%	71.9	17.5	10.6		
10	Receiving organs and tissue donated by a person infected with HIV/AIDS	n	127	17	16	2.69	0.64
		%	79.4	10.6	10.0		
11	Receiving blood from a person infected with HIV/AIDS	n	134	20	6	2.80	0.49
		%	83.7	12.5	3.8		
12	Vaginal liquid of a person infected with HIV/AIDS	n	138	13	9	2.81	0.52
		%	86.3	8.1	5.6		
13	Semen of a person infected with HIV/AIDS	n	140	10	10	2.81	0.53
		%	87.5	6.3	6.3		
14	Urine of a person infected with HIV/AIDS	n	70	62	28	2.26	0.74
		%	43.7	38.8	17.5		
15	Tears of a person infected with HIV/AIDS	n	37	93	30	2.04	0.65
		%	23.1	58.1	18.8		
16	Mucus or nasal fluid of a person infected with HIV/AIDS	n	63	72	25	2.24	0.70
		%	39.4	45.0	15.6		
17	Breast milk of a person infected with HIV/AIDS	n	77	57	26	2.32	0.74
		%	48.1	35.6	16.3		
Total score of knowledge about the routes of transmission of HIV/AIDS (mean ± SD)						41.66	5.61

**Table 4:** Participants’ attitude toward patients with AIDS Data are presented as mean ± SD and n (%).

Item No.	Questions		Agree	Disagree	Mean	SD
1	Dental team with AIDS should assist in special clinics for those with AIDS	n	97	63	1.61	0.49
		%	60.5	39.4		
2	I would not sit at the same desk with a person with AIDS.	n	37	123	1.23	0.42
		%	23.1	76.9		
3	People infected with HIV should be isolated in a special center	n	79	81	1.49	0.50
		%	49.4	50.6		
4	Everybody must know about those with AIDS by means of national media	n	74	86	1.46	0.50
		%	46.2	53.8		
5	People living with HIV/AIDS must be supported, treated, and helped	n	150	10	1.94	0.24
		%	93.7	6.3		
6	I am concerned that working with AIDS patients may endanger my health	n	85	75	1.53	0.50
		%	53.1	46.9		
7	My professional education has provided me with enough information to work safely with AIDS patient	n	139	21	1.87	0.34
		%	86.9	13.1		
8	I am concerned that in the future we will find that AIDS can be transmitted in ways now thought to be safe		112	48	1.70	0.46
		%	70.0	30.0		
9	I am willing to perform mouth-to-mouth resuscitation on an AIDS patient in respiratory arrest	n	52	108	1.33	0.47
		%	32.5	67.5		
10	I would inform an AIDS patient’s sexual partner against the patient’s wishes.	n	103	57	1.64	0.48
		%	64.4	35.6		
11	I believe I have the right to refuse to assist in the treatment of an AIDS patient	n	64	96	1.40	0.49
		%	40.0	60.0		
12	I would refuse to assist in the treatment of an AIDS patient	n	32	128	1.20	0.40
		%	20.0	80.0		
Total score of attitude toward people with AIDS (mean ± SD)					18.4	2.61

In Table 1, participants' knowledge about HIV/AIDS is summarized in frequencies, percentages, mean, and SD. It is noted that the majority of participants (n=153; 95.6%) correctly identified that AIDS is caused by a virus, and about 120 participants (75%) were aware that HIV-positive individuals may appear healthy. In addition, 123 (76.9%) participants agreed that HIV-positive individuals had lower resistance to other diseases compared to healthy individuals. On the other hand, when participants were inquired about anti-HIV antibodies, 55 (34.4%) participants didn’t provide the correct answer, and when asked about the average time between contracting HIV and the production of antibodies, 91 (56.9%) participants did not have the correct response. These findings may suggest that participants' general knowledge about HIV/AIDS may be considered good, yet they may lack in-depth understanding.

Participants' awareness of HIV-associated oral and periodontal lesions is presented in Table 2. More than half of the participants are well-informed about the connections between HIV and conditions such as oral candidiasis, herpes simplex virus, and necrotizing gingivitis, with awareness percentages of 78.8% (126 participants), 70.6% (113 participants), and 63.1% (101 participants), respectively. In Table 3, participants' knowledge regarding the transmission routes of HIV is detailed. The total score is 41.66 ± 5.61, which suggests that participants demonstrated a high level of knowledge regarding the transmission routes of HIV/AIDS. The results in Table 4 present the participants' attitudes toward patients with AIDS. A significant number of participants (n=150; 93.7%), expressed their willingness to treat them, while only 10 (6.3%) participants did not agree to support or treat these patients. Furthermore, the majority of dental hygienists and assistants (n=139; 86.9%) agreed that their professional education had provided them with sufficient knowledge to provide safe care for AIDS patients.

Pearson's correlation analysis revealed a weak and statistically insignificant correlation between the overall scores of participants' general knowledge about HIV/AIDS, knowledge of HIV-associated oral and periodontal lesions, and knowledge about the routes of transmission of HIV with total attitude scores (r = -0.0043, p-value = 0.9568; r = 0.0424, p-value = 0.5938; r = 0.0866, p-value = 0.2764, respectively). The analysis of significant differences based on job roles (dental assistant or dental hygienist) is summarized in Table 5. A t-test was performed, and the results suggest that there are no statistically significant differences between dental hygienists and dental assistants concerning participants' knowledge and attitudes toward HIV/AIDS.

**Table 5:** Comparison between dental assistants and dental hygienists regarding their knowledge and attitude towards HIV/AIDS

Dimensions	Total scores mean by Job Role				T-Test Statistics	P-value
	Hygienist		Assistant			
	Total scores mean	SD	Total scores mean	SD		
Knowledge about HIV/AIDS	23.1	2.59	23.2	2.89	-0.11499	0.9087
Knowledge of HIV-associated oral and periodontal lesions	24.4	4.79	24.2	6.46	0.28009	0.7799
Knowledge about the routes of transmission of HIV/AIDS	40.9	5.64	42.0	5.58	-1.1867	0.2383
Attitude toward patients with AIDS	18.2	2.51	18.5	2.66	-0.53296	0.5952
Tabulated t-value was set at the (0.05) significant level						

## Discussion

The percentage of HIV-positive patients seeking dental treatment has notably increased in the past decade. This could be attributed to the extended life expectancy associated with the widespread use of ART. Moreover, this has also increased self-awareness of health and interest in oral care [36]. Consequently, dental professionals including dental hygienists and assistants are increasingly likely to encounter HIV-infected patients in their daily practice. It is therefore essential to improve strategies that meet the needs of this patient population.

Our study was conducted to assess the level of knowledge and attitude of dental hygienists and dental assistants towards HIV/AIDS patients in Jeddah province, Saudi Arabia. While evaluating dentists' and dental students' knowledge and attitudes toward AIDS patients is a previously explored topic, our study represents the most recent study in Jeddah to specifically focus on the knowledge and attitudes of dental hygienists and dental assistants concerning HIV. In the current study, it's noteworthy that 73.1% (117 individuals) of the participants were females, resulting in a significant gender difference. As a result, it was not feasible to evaluate gender-based differences in knowledge and attitude. However, a previous study conducted on Saudi healthcare workers assessed gender differences and reported that males exhibited superior knowledge and displayed a more positive attitude toward AIDS patients [37].

The provision of suitable dental treatment to HIV/AIDS patients dictates a comprehensive knowledge in the identification of the oral lesions interrelated to the disease. Several oral signs associated with HIV infection have been described [38], and oral lesions often constitute some of the earliest and most reliable signs of HIV [39]. Our study's results indicate that participants demonstrated an adequate level of knowledge regarding lesions commonly observed in association with HIV infection, such as oral candidiasis, herpes simplex virus, and necrotizing gingivitis. However, dental hygienists and assistants need to have a broad knowledge of lesions that are not commonly associated with HIV such as oral Kaposi’s sarcoma, salivary gland enlargement, non-Hodgkin’s lymphoma, and Crohn’s disease. Furthermore, they should be knowledgeable about lesions that may not be exclusive to AIDS patients, such as hairy leukoplakia and oral candidiasis. A study by Oliveira et al. focused on dental students' knowledge and concluded that most of them could effectively diagnose oral lesions in AIDS patients, including conditions like oral hairy leukoplakia, oral candidiasis, and Kaposi's sarcoma [30]. Similarly, the overall knowledge of dental hygienists and assistants about the oral and periodontal lesions associated with HIV/AIDS is deemed satisfactory when compared to dentists and dental students in other countries [40-43]. Moreover, this study highlights the ability of dental hygienists and assistants to identify the relationship between HIV-related immunosuppression and periodontal conditions such as necrotizing gingivitis (101 participants, 63.1%) and aggressive periodontitis (92 participants, 57.5%). Dental hygienists and assistants are instrumental in early HIV detection, recognizing key oral and periodontal lesions, thus enabling timely medical referrals, and advocating for an integrated dental care approach [40].

Although it was not uncommon for many dentists to refuse to provide dental care for HIV patients in the past, there has been a notable shift in dentists' attitudes towards managing these patients in recent years [27]. In our study, only 37 (23.1%) dental hygienists and assistants were unwilling to interact with HIV/AIDS patients and 32 (20%) declined to treat these patients. Interestingly, 79 (49.4 %) participants believed that HIV patients should be treated in dedicated centers. They recognized the importance of providing dental treatment to this population at dental centers but indicated the need for special precautions, anticipating that new modes of disease transmission might emerge in the future. Although these results are in agreement with previous reports on dental professionals' attitudes [31,32,44], the willingness of dental hygienists and assistants to provide care for HIV/AIDS patients might be seen as somewhat optimistic in a conservative culture where the prevalence of HIV remains relatively low. The study also revealed that a significant majority of participants had negative responses when asked about their willingness to work closely with a person with AIDS or to assist in the treatment of an AIDS patient. A study carried out in Jordan, a neighboring country to Saudi Arabia with a similar culture, aimed to explore the willingness of dentists to treat HIV-infected patients. Interestingly, when a simulated HIV patient communicated with dental offices by phone seeking treatment for dental pain, only 15% of dentists approved to provide such care [45].

Dental professionals are vulnerable to risks from blood-borne pathogens during procedures involving exposure to blood and saliva. It is thus imperative for them to have a comprehensive understanding of these pathogens, consistently implement protective barriers, meticulously observe cross-infection preventive measures, and strictly follow sterilization protocols [40]. The current evidence shows a low risk of HIV infection among healthcare professionals [46]. The risk linked with occupational exposure to HIV with needle-stick injury has been estimated to be 0.2-0.5% [47]. Approximately 123 (76.9 %) participants correctly recognized that contact with the intact skin of an HIV patient does not lead to disease transmission. Moreover, 104 (65%) participants correctly identified that HIV is not transmitted by sharing public toilets and swimming pools. This suggests that the sample in this study possesses a good knowledge of the various routes of transmission of HIV, particularly recognizing the risk of infection associated with infected needles or surgical devices, exposure to semen or vaginal fluids from an HIV-infected individual, and receiving blood or tissues from HIV patients.

The study did not inquire about the potential transmission of HIV via saliva, as there is a difference of opinion in the literature. Baron et al. suggested that HIV transmission through saliva is very rare, as the saliva of HIV-infected individuals usually has non-infectious components of HIV [48]. However, it's important to acknowledge that a few cases have reported HIV transmission from patients to dental healthcare workers, with one case involving a woman after receiving an invasive dental procedure [49]. Addressing blood-borne pathogen transmission effectively necessitates a multifaceted strategy, including immunization, preventive measures, and post-exposure interventions. Avoiding exposure remains the primary protective measure against most blood-borne pathogens [47]. Furthermore, ensuring dental professionals are well-versed in post-exposure procedures is critical, enhancing safety for both healthcare providers and patients.

When comparing the knowledge about the nature of the AIDS virus among dental assistants and dental hygienists, dental hygienists exhibited a slightly higher level of knowledge than assistants. However, this difference was not statistically significant. Moreover, the study results demonstrated low correlation coefficients between participants' knowledge levels with their overall attitudes suggesting that variations in knowledge were not strongly associated with corresponding variations in their attitudes toward HIV/AIDS. These findings underscore the importance of considering multiple factors beyond knowledge alone in shaping attitudes towards HIV/AIDS, urging a more comprehensive approach to educational interventions aimed at fostering positive attitudes and reducing stigma surrounding the disease.

One of the primary limitations of our study is that the results rely on subjective assessments due to the questionnaire-based approach. We could not observe real-life behaviors or the practical application of knowledge directly. Additionally, participants were selected through convenient sampling from a single city, which limits the generalizability of the results to the broader population of dental practitioners in Saudi Arabia. Future research may explore the impact of continuous educational dental programs focused on HIV on dental hygienists and assistants' knowledge, attitudes, and practices.

## Conclusions

The dental hygienists and assistants who participated in this study exhibited a comprehensive overall knowledge of HIV and AIDS, including a good understanding of the routes of disease transmission. Furthermore, participants generally displayed positive attitudes toward HIV patients, advocating for their treatment and support rather than isolation. Both dental hygienists and assistants demonstrated a substantial understanding of the link between HIV-induced immunosuppression and various oral and periodontal conditions, recognizing the association with conditions such as oral hairy leukoplakia, necrotizing gingivitis, and aggressive periodontitis. It's important to note that these findings may carry a degree of potential overestimation due to the questionnaire-based nature of the study. Future research might explore the significant effects of continuous, targeted HIV education programs, investigating their role in shaping the knowledge, attitudes, and procedural skills of dental hygienists and assistants, thereby enhancing this vital healthcare area.
